# Significant impairment of health-related quality of life in mainland Chinese patients with chronic hepatitis B: a cross-sectional survey with pair-matched healthy controls

**DOI:** 10.1186/1477-7525-12-101

**Published:** 2014-06-14

**Authors:** Guihua Zhuang, Min Zhang, Yong Liu, Yaling Guo, Qian Wu, Kaina Zhou, Zhenhao Ji, Xiaomei Zhang

**Affiliations:** 1 Department of Epidemiology and Biostatistics, School of Public Heath, Xi’an Jiaotong University Health Science Center, No. 76 West Yanta Road, Xi’an, Shaanxi 710061, China; 2 Xi’an Jiaotong University Health Science Center, No. 76 West Yanta Road, Xi’an, Shaanxi 710061, China; 3 The Eighth Hospital of Xi’an, No. 2 East Zhangba Road, Xi’an, Shaanxi 710061, China

**Keywords:** Chronic hepatitis B, Health-related quality of life, Short Form 36 version 2, Chronic Liver Disease Questionnaire, Preference, Mainland Chinese

## Abstract

**Objective:**

Few studies have evaluated health-related quality of life (HRQoL) of patients with chronic hepatitis B (CHB) in mainland China. We aimed at characterizing the impact of CHB on HRQoL in mainland Chinese and finding out factors associated with HRQoL.

**Methods:**

460 CHB patients (323 with CHB only, 54 with compensated cirrhosis and 83 with decompensated cirrhosis) and 460 pair-matched healthy controls were recruited in Xi’an city. They answered a structured questionnaire including the Short Form 36 version 2 (SF-36v2), the Chronic Liver Disease Questionnaire (CLDQ) (only for patients), and questions on socio-demographic and clinical characteristics. A blood sample was collected from each of patients for liver function tests. SF-36v2 scores were compared between patients and controls overall and by groups by paired-samples t-test, and CLDQ scores and paired differences of SF-36v2 scores were compared among three patient groups by one-way ANOVA or Kruskal-Wallis test. Multi-variable linear regression analyses were performed to identify determinants of HRQoL in patients.

**Results:**

Patients, overall and by groups had significantly lower SF-36v2 scores than controls on all summaries and domains, with differences higher than the suggested minimally important difference values. Both the SF-36v2 and the CLDQ showed that HRQoL of patients with cirrhosis further deteriorated, but compensated and decompensated cirrhosis patients had similar total HRQoL impairments. The gradually increasing impairment with disease progression was confirmed only on physical components. Impaired liver function and currently taken anti-viral treatment were associated with lower HRQoL. Education attainment and annual per capita household income had a positive effect on HRQoL.

**Conclusions:**

Mainland Chinese CHB patients suffered significant HRQoL impairment on all health dimensions, and the impairment reached a high level on mental health at initial stage of illness and increased gradually on physical health with disease progression. Attention should be paid to the reduction of patients’ treatment cost burden and the provision of early health education accompanied with proper treatments.

## Background

Chronic hepatitis B (CHB), caused by persistent infection with hepatitis B virus (HBV), is a common chronic liver disease in mainland China. The latest nationwide serosurvey showed that 7.2% of the population aged 1–59 years were positive for HBV surface antigen [1]. Estimated current HBV carriers run up to 93 million, including 20–30 million patients with CHB [2], which accounts for almost one third of the global total [3]. CHB patients have to cope with many recurrent symptoms in their long disease histories and are at high risk to develop fatal complications of cirrhosis and hepatocellular carcinoma [4]. Thus, CHB may result in a heavy disease burden not only in premature death but in health impairment.

As a multidimensional construct describing individuals’ perceptions of their physical, psychological and social functioning, health-related quality of life (HRQoL) can holistically assess health outcomes than clinical parameters, particularly important in chronic diseases, to help healthcare workers to understand patients’ needs and provide quality health services [5,6]. With growing interest in HRQoL in recent years, there have been some studies focusing on HRQoL of CHB patients [7-11]. A study from Singapore indicated that HRQoL was similar in asymptomatic HBV carriers and normal controls, but obviously decreased in CHB patients and further deteriorated with disease progression [7]. Another from Hong Kong of China found that all individuals with chronic HBV infection had significantly lower HRQoL than general population even among those without any biochemical and clinical abnormalities [8]. Impaired HRQoL in CHB patients with or without cirrhosis was also reported from several other countries [9-11]. However, few studies have come from mainland China. The impact of disease on HRQoL may vary from population to population because of differences in socioeconomic status, cultural heritage, religion and life style [12], just like the differences presented among the previous studies in the impaired dimensions and degrees and the factors associated with the impairment [7-11].

HRQoL measured by a generic instrument with general public’s preference scores can be easily converted to a preference index for use in cost-effectiveness evaluation of healthcare interventions [13]. Up to now, only one study touched upon mainland Chinese preferences for CHB-related health states [12], in which the standard gamble method was used and 100 CHB patients and 100 uninfected individuals were enrolled. Country or region-specific preference estimates may benefit local economic evaluations.

The objective of this study was to characterize the impact of CHB on HRQoL in mainland Chinese and to find out factors associated with the impairment of HRQoL. In addition, we would like to obtain preference estimates of different stages of CHB for use in relevant economic evaluation studies.

## Methods

### Subjects and data collection

A cross-sectional survey was conducted from April to July 2010 in Xi’an city, located in the central-northwest of mainland China with 8.5 million resident population (over 98% are Han Chinese). Its per capita gross domestic product and HBV prevalence are around the national average levels.

CHB patients were selected from outpatients from April to June 2010 and discharged patients from November 2008 to March 2010 of the Eighth Hospital of Xi’an based on their primary diagnoses of CHB or CHB-related compensated or decompensated cirrhosis in medical records. The hospital is the only one specializing in infectious diseases locally, with the most number of outpatient visits and admissions for viral hepatitis. Patients must be Han Chinese aged 18 years or elder and have lived in Xi’an for five years or more. CHB diagnosis and its classification followed the standards in the Guideline of Prevention and Treatment for Chronic Hepatitis B (China, 2005), which are consistent with international consensus [4,7]. Decompensated cirrhosis was defined as cirrhosis with a history of ascites, variceal bleeding, or hepatic encephalopathy. For discharged patients, if the diagnosis for CHB was updated after discharge by a hospital specialist the relevant medical record would be consulted. Patients were excluded if they had any other chronic diseases (e.g. hypertension, heart disease, stroke, diabetes mellitus, chronic lung disease, alcoholic or drug-induced liver disease, tuberculosis, psychological illness) or co-infections (hepatitis C, hepatitis D or human immunodeficiency virus) identified by medical record review or the questionnaire survey; or severe cognitive impairment identified by the inability to understand informed consent.

Patients received an individual face-to-face interview administered by one of trained interviewers in the hospital or in their homes. They answered a structured questionnaire that comprised of the Chinese (mainland) Short Form 36 version 2 (SF-36v2), the Chinese (mainland) Chronic Liver Disease Questionnaire (CLDQ) and questions on socio-demographics, the history of CHB and related treatment, and other chronic morbidities. Immediately after interview, a blood sample was collected for liver function tests including alanine aminotransferase (ALT), total bilirubin (TBIL) and albumin (ALB), which are the most commonly used biomarkers reflecting hepatocellular injury, liver excretory function and liver synthetic function, respectively [14].

A healthy control with matching sex, age (±5 years) and occupation was selected for each patient in his/her resident sub-district by convenient sampling. The inclusion and exclusion criteria above were followed. Controls answered the same questionnaire administered like for patients, but without the questions of CLDQ. Liver function tests were not provided for controls. The study protocol was reviewed and approved by the Human Research Ethics Committees of Xi’an Jiaotong University and the Eighth Hospital of Xi’an, respectively. The written informed consent was obtained from each recruited subject before the questionnaire survey.

### Instruments and outcome measures

#### *Chinese (mainland) SF-36v2*

The SF-36v2 is a generic HRQoL measure which has been widely used in general and specific populations throughout the world [15]. It measures eight health domains including physical functioning (PF), role-physical (RP), bodily pain (BP), general health (GH), vitality (VT), social functioning (SF), role-emotional (RE), and mental health (MH). The eight domains are aggregated into two summary measures: physical component summary (PCS) and mental component summary (MCS). The standard (4-week recall) Chinese (mainland) form was provided by QualityMetric Incorporated, which has been validated in mainland Chinese CHB patients [16]. We scored all domains and summaries as norm-based scores which have the same general population mean of 50 and standard deviation of 10. The scoring was performed by Health Outcomes™ Scoring Software 2.0 (QualityMetric Incorporated), and the general U.S. population norms embedded in the software were used as references due to the lack of general mainland Chinese population norms. For all domains and summaries, higher scores indicate better HRQoL and scores (on group-level) less than 47 indicate being below the average range for the U.S. general population [15].

#### *SF-6D*

Although the SF-36v2 was not originally designed for preference measurement, its 11 items were combined to form the SF-6D, a preference-based measure for deriving a preference index scored from 0 (death) to 1 (full health) [13,17]. The SF-6D scoring model for Hong Kong Chinese population has been established and validated by McGhee et al. [18]. We converted SF-36v2 data to SF-6D preference values using this model.

#### *Chinese (mainland) CLDQ*

The CLDQ developed by Younossi et al. is a commonly used specific HRQoL measure for liver diseases [19]. It consists of 29 items measuring six health domains on abdominal symptoms (AB), fatigue (FA), systemic symptoms (SY), activity (AC), emotional function (EM), and worry (WO). Each item is rated on a 7-point Likert scale. Domain scores are calculated by the summated averages of the respective item scores. An overall score is calculated by the mean of all domain scores. Each domain and the overall score range from 1 to 7, with higher scores indicating better HRQoL. The Chinese (mainland) CLDQ was provided by the developers of the original and it has been validated in mainland Chinese CHB patients [20].

### Data analysis

Patients were considered as a whole, and were also divided into three groups with different stages of illness: CHB, compensated cirrhosis, and decompensated cirrhosis. One-way ANOVA or Pearson’s chi-square test was used to compare differences in socio-demographic and clinical characteristics among three patient groups. Paired-samples t-test was used to compare differences in SF-36v2 and SF-6D scores between patients and controls. Paired differences of SF-36v2 and SF-6D scores were calculated, and they and CLDQ scores were compared among three patient groups using one-way ANOVA or Kruskal-Wallis test. If significant differences were found, pairwise multiple comparisons were conducted to further examine differences between groups. Multi-variable linear stepwise regression analyses were performed to identify factors associated with HRQoL scores in patients. Independent variables in each regression analysis were same, including stage of illness and all socio-demographic and clinical characteristics shown in Table 1. All data analyses were carried out in SPSS 13.0, with *P* value less than 0.05 as statistical significant level.

**Table 1:** Socio-demographic and clinical characteristics of chronic hepatitis B patients

	**Overall (n = 460)**	**Chronic hepatitis B (n = 323)**	**Compensated cirrhosis (n = 54)**	**Decompensated cirrhosis (n = 83)**
Age (years, mean ± SD)^†^	35.8 ± 12.8	31.5 ± 10.9	43.0 ± 10.3	47.4 ± 11.7
Sex (%)				
Male	66.3	65.6	72.2	65.1
Female	33.7	34.4	27.8	34.9
Occupation (%)^†^				
Peasants	33.3	22.6	48.1	65.1
Service and commercial workers	17.0	18.9	14.8	10.9
Enterprise and institution staff	13.3	14.9	14.8	6.0
Unemployees	13.2	16.1	5.6	7.2
Self-employed persons	8.0	9.3	7.4	3.6
Students	7.4	10.5	0.0	0.0
Others	7.8	7.7	9.3	7.2
Education attainment (%)^†^				
No schooling and primary	13.7	9.6	20.4	25.3
Secondary	67.6	67.2	66.7	69.9
Tertiary	18.7	23.2	12.9	4.8
Marital status (%)^†^				
Married	71.3	61.9	96.3	91.6
Other marital status	28.7	38.1	3.7	8.4
Annual per capita household income (Chinese $, %)^†^				
<5000	51.3	45.5	61.1	67.5
5000–9999	24.1	27.3	18.5	15.7
≥10000	24.6	27.2	20.4	16.8
Duration of illness (years, mean ± SD)	6.1 ± 6.9	6.0 ± 6.5	6.4 ± 6.7	6.3 ± 8.2
Currently taken anti-viraltreatment (%)^†^				
No	61.1	67.2	50.0	44.6
Yes	38.9	32.8	50.0	55.4
Liver function tests*				
ALT (U/L, mean ± SD)	61.9 ± 95.0	62.1 ± 100.2	52.4 ± 40.9	70.4 ± 83.1
TBIL (umol/L, mean ± SD)^†^	19.7 ± 20.3	16.5 ± 7.3	24.8 ± 13.7	46.7 ± 60.4
ALB (g/L, mean ± SD)^†^	44.1 ± 4.8	45.0 ± 4.0	42.7 ± 4.7	36.0 ± 4.6

ALT, alanine aminotransferase; TBIL, total bilirubin; ALB, albumin.

*Only including 375 patients receiving liver function tests: 311, 34 and 30 in chronic hepatitis B, compensated cirrhosis and decompensated cirrhosis groups, respectively.

^†^Significant difference among three patient groups (*P* < 0.05).

## Results

### Accomplishment of survey and quality of data

A total of 934 discharged patients within the definite period of time met the inclusion criteria and had no diagnosis of any other chronic diseases or co-infections in their medical records. Of them, 323 could not be contacted due to the absence or change of their telephone numbers and 25 had died. Among 586 contacted patients, 263 refused the interview (132 were busy; 38 were not interested; 31 had health problems; and 62 did not give any reasons), 69 were excluded because they clearly reported their histories and current treatments of other chronic diseases, and 254 finished the questionnaire. A total of 206 outpatients fulfilling the inclusion and exclusion criteria were approached, and they all answered the questionnaire. Therefore, a total of 460 patients were enrolled in this study, including 323 with CHB, 54 with compensated cirrhosis and 83 with decompensated cirrhosis. However, only 375 patients (311, 34 and 30 in the corresponding patient groups above) provided blood samples. Along with the enrollment of patients, 460 pair-matched healthy controls were recruited.

All 920 questionnaires were answered completely, without any missing questions. For the SF-36v2, the quality of data was evaluated by the scoring software and all the seven quantitative checks (completeness of data, responses outside of range, consistent responses, percentage of estimable scale scores, item internal consistency, item discriminant validity, and scale reliability) were within acceptable ranges suggested by QualityMetric [15].

### Patients’ characteristics

Table 1 showed socio-demographic and clinical characteristics of patients overall and by groups. There were significant differences in these characteristics among three patient groups except sex, duration of illness and ALT level. The duration of illness was defined as the self-reported time from the first diagnosis for CHB by a registered medical practitioner to the day of the interview.

The socio-demographic characteristics besides those for matching, i.e. education attainment, marital status and annual per capita household income, were also comparable between patients and controls overall and by groups (*P* > 0.05).

### SF-36v2 scores

**Figure 1:**
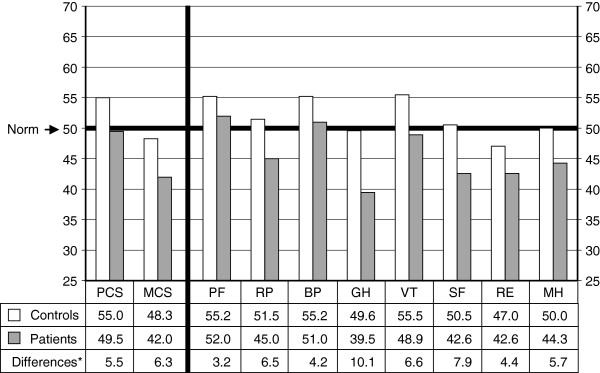
**Mean norm-based scores of SF-36v2 health domains and component summary measures in all 460 chronic hepatitis B patients compared with pair-matched healthy controls.** PCS, physical component summary; MCS, mental component summary; PF, physical functioning; RP, role-physical; BP, bodily pain; GH, general health; VT, vitality; SF, social functioning; RE, role-emotional; MH, mental health. *All differences are statistically significant (*P* < 0.001).

**Figure 2:**
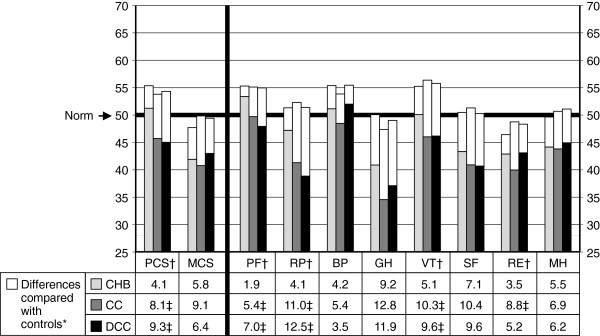
**Mean norm-based scores of SF-36v2 health domains and component summary measures by patient groups, compared with respective pair-matched healthy controls.** CHB, chronic hepatitis B; CC, compensated cirrhosis; DCC, decompensated cirrhosis; PCS, physical component summary; MCS, mental component summary; PF, physical functioning; RP, role-physical; BP, bodily pain; GH, general health; VT, vitality; SF, social functioning; RE, role-emotional; MH, mental health. *All differences are statistically significant (*P* < 0.05). †Significant difference among three patient groups (*P* < 0.05). ‡Significantly different compared with CHB group (*P* < 0.05).

Figure 1 demonstrated the SF-36v2 profile of scores for all patients compared with controls. Controls had mean scores higher than or equal to 47 on all summaries and domains, but patients less than 47 on MCS and RP, GH, SF, RE and MH. Patients scored significantly lower than controls on all summaries and domains (*P* < 0.001), with differences of means (i.e. average paired differences) from 3.2 to 10.1 which were all greater than the suggested minimally important difference (MID) values by QualityMetric, commonly 2 to 3 for group-level comparisons [15].Figure 2 further showed that three patient groups scored significantly lower than respective controls on all summaries and domains. All average paired differences but 1.9 on PF of CHB group were greater than the suggested MID values. Comparing these average paired differences among three patient groups, CHB group was the lowest on all summaries and domains except BP, significantly different from any (or one) of two other groups on PCS and half of the domains. However, no any significant differences were found between compensated and decompensated cirrhosis groups. With disease progression from CHB to compensated cirrhosis and to decompensated cirrhosis, the average paired differences on PCS and on PF and RP, both have the greatest physical factor content among the eight domains, gradually increased. This trend did not appear on MCS and any domains which contribute more to the scoring of MCS (i.e. MH, RE, ST and VT).

### SF-6D values

As shown in Table 2, patients overall and by groups had significantly lower SF-6D values than respective controls (*P* < 0.001), decreasing by 12.26% (0.095/0.775) overall and by 9.78% (0.075/0.767), 19.52% (0.154/0.789) and 16.62% (0.132/0.794) in CHB, compensated cirrhosis and decompensated cirrhosis groups, respectively. Average paired differences were significantly different between CHB group and any of two other groups (*P* < 0.01), but not between compensated and decompensated cirrhosis groups.

**Table 2:** SF-6D values (mean ± SD) of chronic hepatitis B patients compared with pair-matched healthy controls

	**Overall (n = 460)**	**Chronic hepatitis B (n = 323)**	**Compensated cirrhosis (n = 54)**	**Decompensated cirrhosis (n = 83)**
Controls	0.775 ± 0.128	0.767 ± 0.128	0.789 ± 0.122	0.794 ± 0.130
Patients	0.680 ± 0.122	0.692 ± 0.120	0.635 ± 0.106	0.662 ± 0.131
Differences^*†^	0.095 ± 0.182	0.075 ± 0.181	0.154 ± 0.153^‡^	0.132 ± 0.192^‡^

^*^All differences are statistically significant (*P* < 0.001).

^†^Significant difference among three patient groups (*P* = 0.001).

^‡^Significantly different compared with chronic hepatitis B group (*P* < 0.01).

### CLDQ scores

CLDQ scores of patients overall and by groups were shown in Table 3. CHB group among three patient groups scored the highest on overall score and all domains except WO, significantly different from any (or one) of two other groups on overall score and half of the domains. However, there were no any significant differences between compensated and decompensated cirrhosis groups. Like the results of SF-36v2, increasing HRQoL impairment with disease progression appeared on physical health indicated by decreasing mean scores on AB, SY and AC, but not on mental health (i.e. EM and WO).

**Table 3:** CLDQ scores (mean ± SD) of chronic hepatitis B patients

	**Overall (n = 460)**	**Chronic hepatitis B (n = 323)**	**Compensated cirrhosis (n = 54)**	**Decompensated cirrhosis (n = 83)**
AB^*^	5.5 ± 1.1	5.6 ± 1.1	5.4 ± 1.0	5.1 ± 1.2^†^
FA	4.8 ± 1.1	4.8 ± 1.1	4.6 ± 1.1	4.6 ± 1.2
SY^*^	5.4 ± 0.9	5.5 ± 0.8	5.3 ± 0.8^†^	5.0 ± 0.9^†^
AC^*^	5.5 ± 1.1	5.7 ± 1.0	5.0 ± 1.2^†^	4.8 ± 1.1^†^
EM	4.8 ± 1.1	4.9 ± 1.1	4.7 ± 0.9	4.9 ± 1.1
WO	5.1 ± 1.2	5.0 ± 1.2	4.9 ± 1.1	5.2 ± 1.0
Overall^*^	5.2 ± 0.8	5.3 ± 0.8	5.0 ± 0.7^†^	4.9 ± 0.8^†^

AB, abdominal symptoms; FA, fatigue; SY, systemic symptoms; AC, activity; EM, emotional function; WO, worry.

^*^Significant difference among three patient groups (*P* < 0.05).

^†^Significantly different compared with chronic hepatitis B group (*P* < 0.05).

### Factors associated with HRQoL in CHB patients

The factors selected by the stepwise regression analyses on different summary or overall HRQoL scores were showed in Table 4. Stage of illness reflecting disease progression was associated only with SF-36v2 PCS score, with a negative relationship. Impaired liver function, indicated by higher TBIL levels, was associated with lower SF-36v2 PCS and SF-6D scores. Longer duration of illness was associated with lower SF-36v2 PCS and MCS and CLDQ overall scores. Currently taken anti-viral treatment negatively correlated with SF-36v2 MCS, SF-6D and CLDQ overall scores. The regression analyses using each domain score of SF-36v2 or CLDQ as dependent variable further found that currently taken anti-viral treatment had a significantly negative correlation with almost all the domains which contribute more to the evaluation of mental health, but only a few of the domains which contribute more to the evaluation of physical health.

**Table 4:** Multi-variable linear stepwise regression on HRQoL scores in chronic hepatitis B patients^*^

**Dependent variable**	**Independent variable entered in the model**	**Unstandardized coefficient (95% CI)**	**Standardized coefficient**	***P *** **value**
SF-36v2 (PCS)	Stage of illness	-2.548 (-3.795, -1.302)	-0.212	0.000
	Total bilirubin	-0.060 (-0.096, -0.024)	-0.171	0.001
	Education attainment	1.901 (0.687, 3.116)	0.149	0.002
	Duration of illness	-0.110 (-0.213, -0.008)	-0.102	0.035
SF-36v2 (MCS)	Currently taken anti-viral treatment	-3.895 (-6.147, -1.644)	-0.174	0.001
	Education attainment	2.180 (0.247, 4.112)	0.113	0.027
	Duration of illness	-0.170 (-0.335, -0.006)	-0.104	0.043
SF-6D	Currently taken anti-viral treatment	-0.041 (-0.066, -0.016)	-0.164	0.001
	Education attainment	0.025 (0.003, 0.048)	0.117	0.029
	Total bilirubin	-0.001 (-0.001, 0.000)	-0.107	0.036
	Age	-0.001 (-0.002, 0.000)	-0.095	0.076
CLDQ (Overall)	Age	-0.013 (-0.020, -0.006)	-0.182	0.000
	Annual per capita household income	0.138 (0.038, 0.238)	0.138	0.007
	Duration of illness	-0.017 (-0.030, -0.003)	-0.131	0.013
	Currently taken anti-viral treatment	-0.203 (-0.375, -0.030)	-0.116	0.022

^*^Only 375 patients with liver function tests are included in the analyses. PCS, physical component summary; MCS, mental component summary. Independent variables include stage of illness (chronic hepatitis B = 1, compensated cirrhosis = 2, decompensated cirrhosis = 3); sex (male = 1, female = 2); occupation (dummy variable); education attainment (no schooling and primary = 1, secondary = 2, tertiary = 3); marital status (married = 1, other marital status = 2); annual per capita household income (<5000 = 1, 5000–9999 = 2, ≥10000 = 3); currently taken anti-viral treatment (no = 1, yes = 2); and other quantitative variables (age, duration of illness, alanine aminotransferase, total bilirubin, and albumin). A variable is entered into the model if the significance level of its *F* value is less than 0.05 and is removed if greater than 0.1.

A few socio-demographic factors also influenced HRQoL scores. Education attainment was positively related to SF-36v2 PCS and MCS and SF-6D scores. Increasing age had a negative effect on CLDQ overall score and SF-6D value. Annual per capita household income was associated only with CLDQ overall score, with a positive relationship.

## Discussion

Although HRQoL of CHB patients has been evaluated in several counties and regions [7-11], the current study from mainland China where has a high HBV prevalence and the largest CHB population in the world may provide important evidence for expanding the knowledge about the impact of CHB on health of people. The pair-matched healthy controls and the exclusion of patients with any other chronic diseases are distinguishing features of the current study, which can better control the influence of confounding factors and accurately characterize the impact of CHB. A generic instrument provides a global assessment and allows for comparisons with other health conditions, while a disease-specific instrument addresses some important HRQoL domains specifically associated with this disease. Both used together may also verify each other’s findings.

Our study found that CHB patients had significantly lower SF-36v2 scores than healthy controls on all summaries and domains regardless of whether with or without cirrhosis. These HRQoL impairments were clinically significant according to the suggested MID values [15]. The similar results were reported by the study from Hong Kong in which the Chinese (Hong Kong) SF-36v2 was used and local general population norms were taken as controls [8]. In this previous study, however, MCS scores in most patient subgroups were similar to that in local general population. The SF-36v2 MCS measures global mental health: a low score indicates frequent psychological distress and social and role disability due to emotional problems [15]. It is inferred that compared with the CHB patients living in Hong Kong where has the similar HBV prevalence but markedly higher socioeconomic status and healthcare level, our CHB patients suffer more serious emotional problems and so more frequent psychological distress and more substantial social and role disability. Although the previous studies from other countries and regions reported impaired HRQoL in CHB patients with or without cirrhosis [7-11], almost all showed the impairment on some but not all dimensions [7-9,11].

Our results from both the SF-36v2 and the CLDQ showed that HRQoL of CHB patients significantly deteriorated with development of cirrhosis, and that the impairment on physical components had a gradually increasing trend with disease progression. These findings were consistent with those in the Hong Kong and the Singapore studies [7,8]. Our study did not find any significant differences in HRQoL impairment between compensated and decompensated cirrhosis patients. Small sample sizes in both groups might be an explication. More possibly, however, it suggested similar impairment between the two types of patients. The serious complications of ascites, variceal bleeding or hepatic encephalopathy may be relieved after successful treatment, and the patients classified as with decompensated cirrhosis according to their complication histories may have similar or moderately poorer physical health status than those with compensated cirrhosis in their daily life. In addition, adaptation and positive coping behaviors may lead to a positive response shift in patients’ psychological perceptions [8].

As expected, the SF-6D values also indicated significant health impairment in CHB patients regardless of whether with or without cirrhosis. The Hong Kong study reported the SF-6D values of 0.755, 0.745, 0.720 and 0.701 in CHB patients with uncomplicated disease, impaired liver function, hepatocellular carcinoma and cirrhosis, respectively, and 0.787 in local general population [8]. Our healthy controls had the same level of preference for their health status as Hong Kong general population, but our CHB patients lower than the CHB patients living in Hong Kong. In the only study touching upon mainland Chinese preferences for CHB-related health states [12], with the standard gamble method the preference values elicited from uninfected individuals for CHB and compensated cirrhosis were between 0.7 and 0.8, similar to the Hong Kong’s results but higher than ours. However, the preference values elicited from uninfected individuals for decompensated cirrhosis and elicited from CHB patients for all CHB-related states were from 2.5 to 0.6, obviously lower than the Hong Kong’s and our results. The disparities may be caused by differences in participants and preference evaluation methods [12,13]. In addition, another difference should be noted. In the Hong Kong and our studies, the patients selected from outpatients or past discharged patients reported their perceptions based on their own health status in daily life, when some serious or typical symptoms may be relieved. In the study with the standard gamble method [12], however, the respondents reported their perceptions based on the pre-set health state-specific indications. For example, decompensated cirrhosis is indicated as “sometimes I vomit blood and have to go to the hospital for a blood transfusion and to have a tube placed in my stomach through my nose”. This might induce respondents to give their response to typical symptoms presented only at the onset stage usually with needs of in-hospital treatment. CHB is a chronic disease and the vast majority patients will live with it till death [4]. The onset of typical symptoms is only an episode in the long disease history, though it may repeat. Therefore, the Hong Kong’s and our results should be more suitable for use in the cost-effectiveness evaluation of interventions with long-term or lifelong impacts. There was no significant difference in SF-6D value impairment between compensated and decompensated cirrhosis patients in our study. Whether it means the difference of preference between the two states is negligible in relevant cost-effectiveness evaluations, further studies are needed.

The stepwise regression analyses further confirmed the association of HRQoL and stages of CHB: more advanced stages with lower physical health. TBIL level may be considered as an important biomarker associated with physical health of CHB patients. The Hong Kong study also reported the association of higher TBIL levels with lower SF-36v2 PCS scores [8]. Currently taken anti-viral treatment was found having a negative relationship with HRQoL of CHB patients, especially on mental dimensions. This could be due to side effects of treatment or the selection of the patients who were more ill or anxious for treatment [8]. Previous studies on patients with hepatitis C also found that anti-viral treatment reduced HRQoL initially [21], but an improvement was observed after successful treatment [22]. Education attainment and annual per capita household income were positively related to HRQoL of CHB patients. The two factors were rarely addressed in the previous studies. This finding is particularly significant in mainland China, where most CHB cases occur in rural areas with low socioeconomic status and poor education and the treatment places a heavy economic burden on patients and their families [23], which may hinder patients taking regular treatment and increase patients’ worry [24]. It suggests that reducing treatment cost burden and providing health education might play certain roles in improving HRQoL of CHB patients in mainland China.

There were two major limitations in our study. The study was implemented only in an area that limited the generalizability of the results for whole mainland China. The small sample sizes in compensated and decompensated cirrhosis patients limited the power of the related results for drawing more exact conclusions.

## Conclusions

Mainland Chinese CHB patients suffered significant HRQoL impairment on all dimensions, and the impairment reached a high level on mental health at initial stage of illness and increased gradually on physical health with disease progression. CHB patients with compensated and with decompensated cirrhosis had similar total HRQoL impairment in their daily life. The SF-6D preference values obtained can be used in relevant cost-effectiveness evaluations. In order to improve HRQoL of CHB patients, attention should be paid to the reduction of patients’ treatment cost burden and the provision of early health education accompanied with proper treatments.

## Abbreviations

AB: Abdominal symptoms; AC: Activity; ALB: Albumin; ALT: Alanine aminotransferase; BP: Bodily pain; CHB: Chronic hepatitis B; CLDQ: Chronic Liver Disease Questionnaire; EM: Emotional function; FA: Fatigue; GH: General health; HBV: Hepatitis B virus; HRQoL: Health-related quality of life; MH: Mental health; MID: Minimally important difference; MCS: Mental component summary; PCS: Physical component summary; PF: Physical functioning; RE: Role-emotional; RP: Role-physical; SF: Social functioning; SF-36v2: Short Form 36 version 2; SY: Systemic symptoms; TBIL: Total bilirubin; VT: Vitality; WO: Worry.

## Competing interests

The authors declare that they have no competing interests.

## Authors’ contributions

GHZ conducted the design and statistical analyses and drafted the manuscript. MZ participated in the design, survey and statistical analyses. YL and YLG gave the administrative and material support and conducted the study supervision. QW, ZHJ and XMZ participated in the survey. KNZ gave the technical support and participated in the statistical analyses. All authors read and approved the final manuscript.
